# Anaphylactic Reactions Caused by Nafamostat Mesylate during Hemodialysis before Surgery for Carpal Tunnel Syndrome

**DOI:** 10.1155/2021/1148156

**Published:** 2021-12-29

**Authors:** Yuta Nakamura, Kaoru Tada, Masashi Matsuta, Atsuro Murai, Hiroyuki Tsuchiya

**Affiliations:** Department of Orthopaedic Surgery, Graduate School of Medical Sciences, Kanazawa University, 13-1 Takara-machi, Kanazawa 920-8641, Japan

## Abstract

Nafamostat mesylate (NM) has been used to treat pancreatitis and disseminated intravascular coagulation during hemodialysis (HD). However, there have been some reports of adverse effects related to anaphylactic reactions. We present a case in which anaphylactic reactions caused by NM during preoperative HD caused repeated postponement of surgery for carpal tunnel syndrome. Symptoms including fever, shivering, chills, low blood pressure, tachycardia, nausea, and vomiting appeared during preoperative HD, and surgery was postponed thrice. Initially, the patient was misdiagnosed with sepsis because of elevated C-reactive protein and procalcitonin levels. However, since the symptoms appeared only when NM was administered and disappeared quickly after the administration of NM was terminated, the condition was diagnosed as anaphylactic reactions caused by NM. Therefore, it is essential to consider anaphylactic reactions caused by NM as differential diagnoses, when symptoms, such as fever, are observed during perioperative HD.

## 1. Introduction

Nafamostat mesylate (NM) was developed in Japan as a serine protease inhibitor to inhibit various proteases in the coagulation system, fibrinolytic system, complement system, and trypsin [1]. In Japan, in 1986, it was approved as a therapeutic agent for pancreatitis, and in 1989 as a therapeutic agent for disseminated intravascular coagulation (DIC) and an anticoagulant drug during hemodialysis (HD). However, there are reports of adverse effects related to allergic reactions, including anaphylactic reactions [2–5], apart from other side effects [6,7]. Here, we report a case in which anaphylactic reactions caused by NM occurred during preoperative HD, and surgery for carpal tunnel syndrome was postponed thrice.

## 2. Case Report

A 66-year-old man started HD 20 years ago with end-stage renal disease due to diabetic nephropathy. Numbness of the left thumb, index, and middle fingers appeared a year ago. Numbness of the fingers worsened at night, and Tinel's sign and Phalen's test were both positive. On electrophysiological examination, the right median distal motor latency from the wrist to the abductor pollicis brevis muscle was 10.86 ms, and the right sensory conduction velocity between the index finger and the wrist was undetectable. Based on the symptoms and physical and laboratory findings, the patient was diagnosed with carpal tunnel syndrome on the right side. Surgery was planned as conservative treatment was ineffective. The preoperative blood test results are presented in Table 1. HD was performed 3 times a week for 4 hours each using ABH-21F (Asahi Kasei Medical, Tokyo, Japan) as a hemodiafilter, and heparin as an anticoagulant before admission. The blood flow rate was 200 ml/min, and the dialysate flow rate was 500 ml/min. NM had never been used previously for this patient. The patient was scheduled to be operated upon immediately following HD.

**Figure 1:**
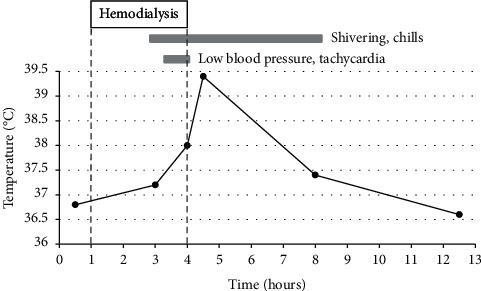
The clinical course of the anaphylactic episode leading to postponement of surgery for a second time.

However, surgery was postponed because a fever of approximately 38°C suddenly appeared during HD before surgery. Surgery was postponed for a week. Once again, a fever of approximately 38°C appeared during HD before the second surgery. Surgery was postponed for a second time, as the patient presented with fever, shivering, chills, low blood pressure, and tachycardia (Figure 1). Physical findings were as follows: height, 168.4 cm; weight, 72.2 kg; systolic blood pressure, 80–90 mmHg; pulse, 108 beats/min. The blood test results are shown in Table 2. DIC was diagnosed based on the DIC diagnostic criteria established by the Japanese Association for Acute Medicine [8], which consists of 8 points (systemic inflammatory response syndrome, 1 point; platelet, 3 points; prothrombin time ratio, 1 point; fibrin degradation product, 3 points). Whole-body computed tomography scan and echocardiography findings were normal, and blood culture was negative; however, sepsis was suspected at first because blood tests indicated an inflammatory response, and procalcitonin levels were high. Meropenem (0.5 g/day) and vancomycin (0.5 g/each HD) were administered. Symptoms, including fever, disappeared a few hours after HD. Although the patient's condition had improved clinically, blood tests performed several days later showed elevated levels of C-reactive protein (CRP) of 4.97 mg/dL and procalcitonin (PCT) of 162 mg/dL, still indicative of sepsis. However, there was no recurrence of symptoms. Subsequently, CRP and PCT levels improved gradually and became negative 17 days after antibiotic treatment; therefore, antibiotic treatment was completed.

One month after antibiotic treatment, surgery for carpal tunnel syndrome was rescheduled. Once again, fever (approximately 38°C), nausea, and vomiting appeared during HD before surgery, and surgery had to be postponed. Symptoms, including fever, disappeared a few hours after HD, as on the previous two occasions. At this point, it was found that the anticoagulant during HD had been changed from heparin to NM only during the perioperative period to control bleeding. The symptoms appeared only when NM was used and disappeared quickly after administration of NM was stopped, leading to the diagnosis of anaphylactic reactions caused by NM. The drug-induced lymphocyte stimulation test (DLST) for NM was negative.

## 3. Discussion

HD requires anticoagulants to prevent coagulation during extracorporeal circulation. Heparin is the most frequently used anticoagulant, but preoperative administration carries a risk of bleeding. In such cases, changing to NM, which has a shorter half-life, is one of the options for controlling bleeding. NM exhibits an anticoagulant effect by inhibiting proteases associated with thrombin and activated coagulation factors (XIIa, Xa, and VIIa). NM has a low molecular weight of approximately 540 Da and is efficiently removed by HD with a half-life of 8 min. Therefore, the anticoagulant effect does not work inside the body but only during extracorporeal circulation [9]; thus, it is used during HD for the perioperative period and bleeding tendencies, such as gastric ulcers and certain conditions such as aortic dissection and heparin-induced thrombocytopenia.

However, side effects must be carefully considered while administering NM. According to the database provided by the Pharmaceuticals and Medical Devices Agency in Japan, there were 2008 cases of side effects caused by NM during the 16 years from 2004 to 2019; of these, 1072 cases (53.4%) were anaphylactic reactions, 45 cases (2.2%) were leukocytosis/leukopenia or thrombocytosis/thrombocytopenia, 43 cases (2.1%) were fever, 29 cases (1.4%) were nausea and vomiting, and 10 cases (0.5%) were shivering and chills. It has also been reported that anaphylactic reactions occur in 0.16% of patients. These reports indicate that side effects related to anaphylactic reactions are among the most common side effects of NM. In the case of anaphylactic reactions caused by NM, it is necessary to note that symptoms may not occur or may be mild at the first use of NM, but may worsen after multiple uses [2, 3].

In this case, CRP and PCT levels were elevated in addition to fever, shivering, chills, and low blood pressure. Since patients on HD are 26 times more likely to suffer from bacteremia than the general population [10], sepsis was strongly suspected at first. However, all culture tests were negative, and imaging tests showed no other lesions that could cause infection or fever. It was also atypical for the course of sepsis as fever and other symptoms disappeared promptly after HD on all occasions. In this case, the DLST for NM was negative. However, although DLST for the drug is widely used for diagnosis, when anaphylactic reactions are suspected, the test has low sensitivity [11]. Another blood test for antigen-specific IgE antibodies also has similar low sensitivity. Therefore, the diagnosis of anaphylactic reactions requires a comprehensive judgment based on clinical symptoms, clinical course, and laboratory findings. Therefore, this patient's condition was diagnosed as anaphylactic reactions caused by NM based on a comprehensive judgment, since DLST for NM was negative, but his condition was stable during HD, except during the perioperative period; symptoms appeared only during HD using NM and disappeared promptly after.

There are several reasons why the diagnosis was delayed in this case. First, despite anaphylactic reactions, PCT, which is known to be specifically elevated in sepsis, was observed. However, it has been reported that 21% of patients with allergy have elevated PCT levels, and PCT levels correlate with the severity of allergic reactions [12]. Therefore, there is a need to pay attention to anaphylactic reactions that mimic bacterial infections. Secondly, the change in anticoagulant during HD was known after a long delay, slowing the diagnosis. This underlines the need for surgeons/physicians to keep in close contact with the dialysis unit.

## 4. Conclusion

We encountered a case of a patient whose surgery for carpal tunnel syndrome was postponed thrice due to acute symptoms, including fever, chills, and tachycardia, during perioperative HD. If such symptoms are observed during perioperative HD, it is necessary to consider anaphylactic reactions to NM as a differential diagnosis.

## Figures and Tables

**Table 1:** Blood test on hospital admission.

WBC	7480/*µ*L
RBC	378 × 10^4^/*µ*l
Hb	11.8 g/dL
Ht	36.8%
Plt	12.4 × 10^4^/*µ*L
TP	5.7 g/dL
Alb	3.5 g/dL
AST	11 IU/L
ALT	7 IU/L
LDH	197 IU/L
T-Bil	0.4 mg/dL
AMY	79 IU/L
CK	65 IU/L
BUN	50 mg/dL
Cr	7.87 mg/dL
UA	6.1 mg/dL
Na	142 mEq/L
K	3.2 mEq/L
Cl	106 mEq/L
CRP	0.33 mg/dL
HbA1c	6.7%
PT	12.5 sec
PT ratio	1.05
APTT	29.5 sec
Fbg	356 mg/dL

**Table 2:** Blood test when a fever appeared for the second time.

WBC	2240/*µ*L
RBC	337 × 10^4^/*µ*l
Hb	10.3 g/dL
Ht	32.3%
Plt	6.4 × 10^4^/*µ*L
TP	5.5 g/dL
Alb	3.2 g/dL
AST	21 IU/L
ALT	12 IU/L
LDH	517 IU/L
T-Bil	0.6 mg/dL
AMY	33 IU/L
CK	36 IU/L
BUN	23 mg/dL
Cr	4.45 mg/dL
UA	3.7 mg/dL
Na	144 mEq/L
K	3.1 mEq/L
Cl	108 mEq/L
CRP	0.66 mg/dL
PT	14.6 sec
PT ratio	1.24
APTT	32.1 sec
Fbg	100.8 mg/dL
D-Dimer	36.9 *µ*g/dL
FDP	75.3 g/dL
PCT	2.9 ng/mL
*β*D-Glucan	<6.0 pg/mL
T-SPOT	–

## Data Availability

The data used to support the findings of this study are included within the article.
